# First-Principles Study of AlPO_4_-H3, a Hydrated Aluminophosphate Zeotype Containing Two Different Types of Adsorbed Water Molecules

**DOI:** 10.3390/molecules24050922

**Published:** 2019-03-06

**Authors:** Michael Fischer

**Affiliations:** 1 Crystallography Group, Department of Geosciences, University of Bremen, Klagenfurter Straße 2-4, D 28359 Bremen, Germany; michael.fischer@uni-bremen.de; 2 MAPEX Center for Materials and Processes, University of Bremen, D 28359 Bremen, Germany

**Keywords:** zeolites, adsorption, density functional theory, molecular dynamics, crystallography

## Abstract

Porous aluminophosphate zeotypes (AlPOs) are promising materials for heat transformation applications using water as a working fluid. Two “types” of adsorbed water molecules can be distinguished in hydrated AlPOs: Water molecules adsorbed in the direct proximity of framework aluminium atoms form bonds to these Al atoms, with the coordination number of Al increasing from four to five or six. The remaining water molecules that are adsorbed in other parts of the accessible pore space are not strongly bonded to any framework atom, they interact with their environment exclusively through hydrogen bonds. The APC-type small-pore aluminophosphate AlPO_4_-H3 contains both types of H_2_O molecules. In the present work, this prototypical hydrated AlPO is studied using dispersion-corrected density functional theory (DFT) calculations. After validating the computations against experimental crystal structure and Raman spectroscopy data, three interrelated aspects are addressed: First, calculations for various partially hydrated models are used to establish that such partially hydrated phases are not thermodynamically stable, as the interaction with the adsorbed water molecules is distinctly weaker than in fully hydrated AlPO_4_-H3. Second, IR and Raman spectra are computed and compared to those of the dehydrated analogue AlPO_4_-C, leading to the identification of a few “fingerprint” modes that could be used as indicators for the presence of Al-coordinated water molecules. Finally, DFT-based molecular dynamics calculations are employed to study the dynamics of the adsorbed water molecules. All in all, this in-depth computational study of AlPO_4_-H3 contributes to the fundamental understanding of hydrated AlPOs, and should therefore provide valuable information for future computational and experimental studies of these systems.

## 1. Introduction

The adsorption of water in aluminophosphate zeotypes (AlPOs) is of particular interest due to their potential application in adsorption-based heat transformations using water as a working fluid [1,2,3]. Moreover, it is also interesting on a more fundamental level, as previous work has shown that two types of adsorbed water molecules can be distinguished in hydrated AlPOs, namely (1) water molecules that are bonded to framework Al atoms, leading to a change in the coordination environment of aluminium to fivefold (trigonal-bipyramidal) or sixfold (octahedral) coordination, and (2) water molecules that reside in the pores, which interact with oxygen atoms of the pore wall or of other water molecules through hydrogen bonds. Experimental evidence for the existence of “Al-coordinated” and “pore” water molecules has been obtained for several AlPOs with varying pore sizes using a portfolio of experimental methods, including NMR spectroscopy [4,5,6,7,8,9], single-crystal X-ray diffraction (sc-XRD) [10], powder X-ray diffraction (PXRD) [8,9,11,12], and vibrational (Raman) spectroscopy [7].

For water molecules coordinated to framework Al atoms, typical Al-O_water_ bond lengths are in the range of 1.95 to 2.05 Å [10,11,12], which is close to the sum of ionic radii of 1.935 Å [13]. The formation of relatively short Al-O_water_ bonds could indicate that the interaction with these water molecules is stronger than for “pore” water molecules that are bonded only through hydrogen bonds. If this was the case, it would imply that partially hydrated AlPOs that contain only Al-coordinated water molecules, but no additional adsorbed water molecules in the pores, should exist as an intermediate phase between the dehydrated and fully hydrated forms. However, joint experimental and computational studies of hydrated AlPOs with medium-sized to large pores like AlPO-34 (CHA topology) [9,12], AlPO-18 (AEI topology) [8], and an LTA-type AlPO [14] have provided no evidence for the existence of such partially hydrated phases. Density functional theory (DFT) calculations for AlPO-34 delivered a binding energy of −14 kJ mol^−1^ for one water molecule per chabazite cage (one five-coordinated Al atom) [14]. For four molecules per cage (two six-coordinated Al atoms), a binding energy of −49 kJ mol^−1^ per H_2_O was calculated, which is still significantly less than the value of −67 kJ mol^−1^ per molecule obtained for a fully hydrated phase with ten H_2_O molecules per cage. Such an increase of the interaction energy with increasing number of adsorbed molecules is contrary to the trend observed in most adsorption processes, where the heat of adsorption decreases with coverage because the energetically most favourable sites are occupied first. It points to a dominant role of water-water interactions. On the basis of these calculations, it was concluded that a complete filling of the pore occurs once the chemical potential of the water vapour is high enough to allow the formation of six-coordinated Al atoms, and that there is no stable intermediate phase that contains only Al-coordinated water molecules. In other words, hydrogen bonds to water molecules in the pores are necessary to stabilise Al-coordinated water molecules in AlPO-34 and, presumably, also in other AlPOs with pores of a similar size. The situation is different in non-porous hydrated aluminophosphates like variscite and metavariscite, where only Al-coordinated water molecules are present, which form hydrogen bonds to framework oxygen atoms [15,16].

AlPO_4_-H3 is a hydrated aluminophosphate zeotype with composition AlPO_4_·1.5H_2_O that was first synthesised by d’Yvoire [17]. Its crystal structure was determined from single-crystal data by Pluth and Smith [10]. The framework, which was assigned the APC framework type code by the International Zeolite Association [18], consists of PO_4_ tetrahedra (atoms P1, P2), AlO_4_ tetrahedra (Al1), and AlO_4_(H_2_O)_2_ octahedra (Al2), with each framework oxygen atom being bonded to one Al and one P atom (i.e., all linkages are Al-O-P linkages). The APC framework can be described as a stacking of $4\cdot 8^{2}$ layers along the *c*-axis, leading to straight channels bordered by eight-membered rings that run along *c*. The channels are interconnected by eight-ring windows along *a*, forming a two-dimensional channel system. The whole structure can be built up from a single building unit (natural tile) with the face symbol $\left[4^{5}\cdot 6^{2}\cdot 8^{3}\right]$ [19].

**Figure 1:**
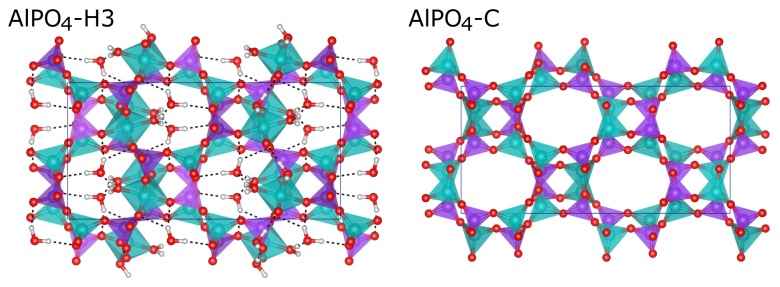
DFT-optimised structures of AlPO_4_-H3 and AlPO_4_-C.

There are two different types of water molecules in the structure of AlPO_4_-H3: In addition to the two Al-coordinated water molecules that are part of the octahedra (called H_2_O(1) and H_2_O(2) throughout this work), a third water molecule H_2_O(pore) is located in the channels that run along *c* (see Figure 1, a more detailed description of the environment of the different water molecules is given below). Dehydration of AlPO_4_-H3 leads to AlPO_4_-C, which has the same topology [17]. The dehydration occurs between 80 and 130 ${}^{\circ }$C, and thermogravimetric measurements show that all water molecules are removed during a single mass loss step [20]. The dehydration is reversible at low temperatures, but heating to about 250 ${}^{\circ }$C leads to a topotactic transformation to AlPO_4_-D (APD framework type) [21]. AlPO_4_-D can in turn be rehydrated to form AlPO_4_-H6. While both the APC and the APD framework can be described as a stacking of $4\cdot 8^{2}$ layers, the chains connecting these layers differ (double-crankshaft chains in APC, nasarsukite-type chains in APD) [21].

This article presents an in-depth computational study of the hydrated aluminophosphate AlPO_4_-H3 by means of dispersion-corrected DFT. The aim of this study is threefold: First, the interaction strength with water in AlPO_4_-H3 and partially hydrated models of APC-type AlPOs is evaluated in order to elucidate the potential (in)stability of partially hydrated phases. Second, the vibrational spectra of AlPO_4_-H3 and dehydrated AlPO_4_-C are predicted using density functional perturbation theory. By identifying modes that are present in the hydrated form, but not in the dehydrated structure, and by analysing the displacement patterns associated with these modes, it is attempted to propose “fingerprint” modes that are indicative for water molecules in different environments. Finally, DFT-based ab initio molecular dynamics (AIMD) calculations are performed to analyse the dynamic behaviour of AlPO_4_-H3, with a particular focus on the dynamics of the water molecules.

## 2. Results

### 2.1. Method Validation

Structure optimisations of fully hydrated AlPO_4_-H3 and dehydrated (calcined) AlPO_4_-C were performed exploiting the symmetry of the experimentally determined structures (AlPO_4_-H3: space group Pbca, ITA No. 61, unit cell content Al_16_P_16_O_64_·24H_2_O; AlPO_4_-C: space group Pbca, ITA No. 61, unit cell content Al_16_P_16_O_64_; in both structures, all atoms are located on general positions with multiplicity 8). Table 1 compares the lattice parameters as well as selected average bond lengths obtained in DFT calculations using the PBE-TS functional [22,23] to experimental values. For the case of AlPO_4_-H3, the most significant deviation in the lattice parameters is an overestimation of the length of *a* by 0.15 Å (0.75%), whereas *b* and *c* agree to within 0.03 Å (0.3%). The lengths of the bonds between framework oxygen atoms and tetrahedrally coordinated Al and P atoms (*d*(Al1-O_fw_), *d*(P-O_fw_)) and octahedrally coordinated Al atoms (*d*(Al2-O_fw_)) are moderately overestimated by 0.015 to 0.025 Å. The same applies for the bonds from octahedrally coordinated aluminium to the water molecules (*d*(Al2-O9), *d*(Al2-O10)). With lengths of 1.981 Å and 1.965 Å, respectively, these bonds are roughly 0.1 Å longer than the average Al2-O_fw_ distance. The general tendency to deliver too long bonds is in line with previous work, where an overestimation of T-O bond distances (T = tetrahedrally coordinated atoms) in all-silica zeolites and aluminophosphates was observed for PBE-based approaches, independent of the presence/absence of a dispersion correction [24]. While this is partly an inherent feature of this functional, it was also shown that the agreement between DFT and experiment can be improved if the experimental bond distances are corrected for the correlated motion of TO_4_ tetrahedra [25]. Indeed, correcting the experimental bond distances using the “simple rigid bond” (SRB) model of Downs et al. [26] brings experimental and computed values closer together (Table 1), but the tendency to overestimate the T-O bond lengths remains prominent.

For the case of AlPO_4_-C, the agreement in lattice parameters is markedly less good than for AlPO_4_-H3, as the length of *a* is overestimated by almost 0.6 Å (∼3%), whereas *b* is underestimated by 0.4 Å (∼4%). For the T-O bond lengths, there is a trend to overestimate these distances by roughly 0.02 Å, as for AlPO_4_-H3. While the average Al-O and P-O bond lengths are essentially the same for AlPO_4_-H3 and AlPO_4_-C, it is worth noting that the scatter in the individual experimental values is considerably larger for the latter system (see Table S1 of Supporting Information), where the structure was refined from powder data.

**Figure 2:**
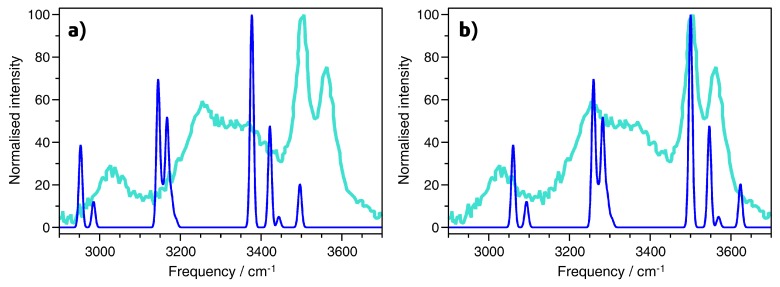
Comparison of DFT-computed Raman spectrum (blue) to experimental data (cyan) [7]. (**a**) Unscaled PBE-TS spectrum. (**b**) PBE-TS spectrum scaled by a factor of 1.037.

A Raman spectrum of AlPO_4_-H3 for the wavenumber range from ∼2900 cm^−1^ to ∼3800 cm^−1^ was reported by Knops-Gerrits et al. [7]. These authors identified a total of five bands, centered at 3012, 3255, 3379, 3505, and 3563 cm^−1^. Figure 2 presents a comparison of the experimental Raman spectrum to the spectrum obtained from PBE-TS calculations for the same wavenumber range, with Figure 2a showing the unscaled calculated spectrum (for the purpose of visualisation, a Gaussian broadening of 10 cm^−1^ was applied to all spectra). The comparison with experiment shows a systematic shift of the PBE-TS bands to lower frequencies, however, the relative positions and intensity ratios are reproduced rather well. An excellent match between the DFT-computed and experimental Raman spectrum can be obtained by scaling the frequencies by a common factor of 1.037, as shown in Figure 2b. A scaling factor in this range does not seem completely unreasonable for a PBE-based approach [27,28]. For a free water molecule (H_2_O placed in a large box), PBE-TS gives O-H stretching frequencies of 3584 cm^−1^ and 3699 cm^−1^, compared to experimental values of 3657 and 3756 cm^−1^, and scaling by a slightly smaller factor of 1.018 would be necessary to give agreement to within 10 cm^−1^ for both frequencies. For the H-O-H bending mode, however, the PBE-TS frequency is slightly overestimated, with 1607 cm^−1^ compared to an experimental value of 1595 cm^−1^. Thus, no simple scaling would lead to agreement between experiment and DFT across the whole frequency range. Since the main aim of this work is not an accurate quantitative prediction of the frequencies, but a qualitative interpretation of the modes, only unscaled IR and Raman spectra will be shown throughout.

In addition to the PBE-TS calculations, further calculations for AlPO_4_-H3 and AlPO_4_-C were performed using the PBEsol-D2 functional [29]. This functional was found to give very good agreement with experimental structure data for guest-free AlPOs and all-silica zeolites in previous work [25]. While use of PBEsol-D2 resulted in better agreement with experimental lattice parameters than found for PBE-TS, a more pronounced tendency to underestimate the length of the hydrogen bonds was observed (see following section). Moreover, the calculated Raman spectrum agreed less well with experimental data. Therefore, the PBEsol-D2 results are not discussed any further.

### 2.2. Hydrogen Bonds

**Figure 3:**
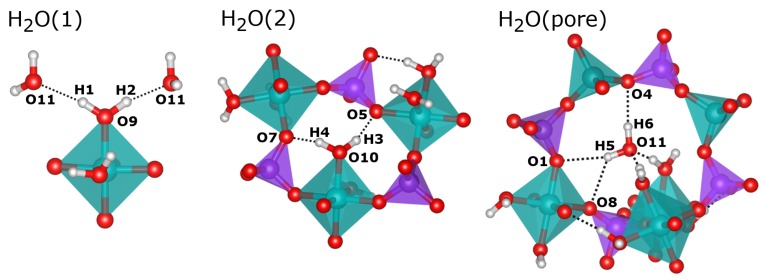
Visualisation of the environment of water molecules in the DFT-optimised structure of AlPO_4_-H3.

In their sc-XRD investigation, Pluth and Smith obtained an almost complete structure of AlPO_4_-H3, which includes the oxygen positions of the three non-equivalent water molecules [10]. The H_2_O(1) and H_2_O(2) molecules are part of the octahedral coordination environment of the Al2 atom. The oxygen atoms of these H_2_O molecules are labelled O9 (H_2_O(1)) and O10 (H_2_O(2)) in this work to maintain consistency with the published crystal structure. The oxygen atom O11, which corresponds to the third water molecule H_2_O(pore), resides near the center of the eight-ring channels. For H_2_O(1) and H_2_O(2), Pluth and Smith also refined the hydrogen positions (H1/H2 and H3/H4), fixing the O-H bond lengths to 0.85 Å and the H-O-H angles to the tetrahedral angle. The H_2_O(1) molecule points into the eight-ring channels that run along the *c*-axis. Its hydrogen atoms, H1 and H2, form relatively long hydrogen bonds (H···O distances of ∼2.1 and ∼2.2 Å) to the oxygen atom O11, i.e., the H_2_O(pore) water molecule (see Figure 3). In contrast, the H_2_O(2) molecule projects into six-rings, and the attached hydrogen atoms H3 and H4 form shorter hydrogen bonds (H···O distances of ∼1.8 and ∼2.0 Å) to two framework oxygen atoms, O5 and O7. The experimental localisation of the hydrogen atoms of the H_2_O(pore) molecule from sc-XRD data was not possible, so these atoms (H5 and H6) were added for the calculations.

A comparison of experimental and DFT-optimised hydrogen bond distances and O-H···O angles is given in Table 2. While the hydrogen bonds formed by the Al-coordinated water molecules have already been described above, those of H_2_O(pore) can only be assessed after the DFT optimisation: Here, the H6 hydrogen atom forms a single hydrogen bond to the framework oxygen atom O4, whereas the H5 hydrogen has similarly close contacts to O1 and O8, both in the range of 2.35 Å (Figure 3). This bonding scenario is best described as a “bifurcated” hydrogen bond, and is reminiscent of some hydrogen bonds present in the nonporous hydrated aluminophosphate metavariscite [15]. For the Al-coordinated water molecules, a comparison of the DFT-optimised H···O distances to experimental values shows that the hydrogen bonds are consistently shorter in the PBE-TS optimised structure, with differences up to 0.36 Å. This can be attributed to the underestimation of hydrogen bond distances by PBE-based approaches, which has been demonstrated previously [30,31,32], and to the use of a fixed covalent O-H bond length of 0.85 Å in the sc-XRD structure refinement, which is considerably smaller than the (more realistic) PBE-TS equilibrium value of 0.99 Å [31]. Indeed, if the O···O distances are compared, experimental and PBE-TS distances agree within ∼0.05 Å for hydrogen bonds from H_2_O(2) and H_2_O(pore) to framework oxygen atoms (this includes the bifurcated H bond). However, agreement is less good for the two hydrogen bonds from H_2_O(1) to H_2_O(pore): Here, one O···O distance is 0.13 Å longer in the PBE-TS structure compared to experiment, whereas the other one is 0.21 Å shorter. A closer inspection reveals that the O11 oxygen atom of H_2_O(pore) moves approximately 0.2 Å along the channel during the DFT optimisation, causing the observed changes of the interatomic distances. As we will see below, when discussing the results of the AIMD simulations, H_2_O(pore) does indeed exhibit a rather large mobility in the channel, and it can thus be expected that the dynamic behaviour affects the average position of this molecule in the real structure at room temperature (the sc-XRD experiment was performed at *T* = 295 K).

### 2.3. Comparison of Fully and Partially Hydrated APC Phases

Table 3 reports the interaction energies $\Delta E_{int}$ and adsorption enthalpies $\Delta H_{ads}$ (for *T* = 298 K) obtained from the DFT calculations for fully hydrated AlPO_4_-H3 and for partially hydrated forms in which either one or two of the three non-equivalent water molecules were removed. There are eight water molecules of each kind in the unit cell. In the partially hydrated structures, all symmetry-equivalent images of the respective water molecule(s) were removed, retaining the Pbca symmetry. Optimised unit cell parameters and structure projections of these partially hydrated phases are given in the Supporting Information. For AlPO_4_-H3, the interaction energy of about −82 kJ mol^−1^ is more negative (i.e., the interaction is stronger) than energies obtained in analogous PBE-TS calculations for six AlPOs with larger pores and different topologies (among them AlPO-34 and AlPO-18), which amounted to approximately −65 kJ mol${}^{-1}$ [33]. The difference can be explained with the ordered arrangement of water molecules in the narrow channels of AlPO_4_-H3, which maximises the number of hydrogen bonds per molecule. In contrast, a disordered distribution of the water molecules was assumed in the calculations for AlPOs with larger pores. It is also worth noting that the relative contribution of non-dispersive interactions is larger in AlPO_4_-H3, amounting to about 70% of the total interaction energy, compared to ∼60 to 65% in other AlPOs, confirming the larger energetic contribution of hydrogen bonds (this contribution was obtained by calculating the difference of the “pure” PBE energies, without the TS dispersion energy, and dividing it by $\Delta E_{int}$). While no enthalpy of water adsorption has been determined experimentally for AlPO_4_-H3, it should be noted that the calculated $\Delta H_{ads}$ of −67 kJ mol^−1^ is considerably more negative than experimental values determined for systems like AlPO-18, which are typically in the range of −55 kJ mol^−1^ [2]. This is in line with the higher temperature of dehydration: Whereas AlPO-18 can be dehydrated below 100 ${}^{\circ }$C [2,34], dehydration of AlPO_4_-H3 occurs between 80 and 130 ${}^{\circ }$C [20]. Thus, both experiment and DFT calculations indicate that AlPO_4_-H3 interacts more strongly with water than AlPOs with larger pores. Nevertheless, it should be emphasised that one cannot expect quantitative accuracy from the calculations, which is why the PBE-TS enthalpy of adsorption should be considered as a semi-quantitative estimate.

For the partially hydrated phases, we observe a rather large variation in the unit cell volumes. For example, the removal of H_2_O(1) leads to a structure in which both the Al1 and Al2 atoms are in trigonal-bipyramidal coordination, causing a significant reduction of the unit cell volume to 1473 Å^3^, whereas the other 2/3 hydrated models have volumes that are much closer to that of AlPO_4_-H3 (∼1800 Å^3^). Nevertheless, the interaction energy—which effectively constitutes a combination of energetic contributions from host-guest interactions and from the overall deformations of the host structure—is very similar for all three 2/3 hydrated models. This is not the case for the 1/3 hydrated phases, where the $\Delta E_{int}$ value of −45 kJ mol^−1^ obtained for the model containing only the H_2_O(1) molecule differs considerably from those of the other two models, which are in the range of −70 kJ mol^−1^. A closer inspection of the structures shows that the water molecules are coordinated to framework Al atoms in all three structures, and that they participate in one or two short hydrogen bonds. The local environment of the water molecule thus provides no apparent explanation for the large difference in the interaction energy. However, the host structure is deformed to a varying extent: In the models containing only H_2_O(2)/H_2_O(pore), pronounced elliptical distortions of eight-rings/six-rings cause a reduction of the unit cell dimensions, with the unit cell volumes being 13/10% smaller than that of AlPO_4_-H3. Such a volume reduction does not happen in the model containing only H_2_O(1). While we observe an inverse correlation between unit cell volume and stability for the 1/3 hydrated phases, a correlation that is well-established for guest-free tetrahedral frameworks [35], this is not found for the 2/3 hydrated models. Altogether, these findings point to a complex interplay of the energetic contributions from hydrogen bonds, from local framework distortions (non-optimal bonding geometries/distorted coordination environments), and from global framework deformations (changes in unit cell volume).

### 2.4. Vibrational Spectra

An analysis of the DFT-computed IR and Raman spectra was performed with emphasis on two different aspects: On the one hand, it was analysed which water molecules are responsible for which bands in the frequency region above 1200 cm^−1^ (O-H stretching, H-O-H bending) in order to test whether vibrational spectroscopy allows for a distinction between Al-coordinated molecules and water molecules adsorbed in the channels. On the other hand, it was attempted to identify lower-frequency modes (<1200 cm^−1^) that could potentially be used as “fingerprint” modes indicative for the presence of six-coordinated Al atoms. The analysis proceeded as follows: After calculating IR and Raman spectra, the displacement patterns associated with all modes with significant intensity were visualised using Jmol [36] in order to get a qualitative overview of the most prominent displacements. In the following, the magnitude of the atomic displacements was analysed in a more quantitative fashion to determine which atoms make the largest contribution to certain modes of particular interest. Clearly, any such assignment remains a simplification, because every mode is associated with the displacement of several atoms. Nevertheless, it was often possible to identify one or a few non-equivalent atom(s) that predominantly contribute to a given mode. The Supporting Information contains the CASTEP *.phonon files of AlPO_4_-H3 and AlPO_4_-C, from which the atomic displacements can be visualised with Jmol.

**Figure 4:**
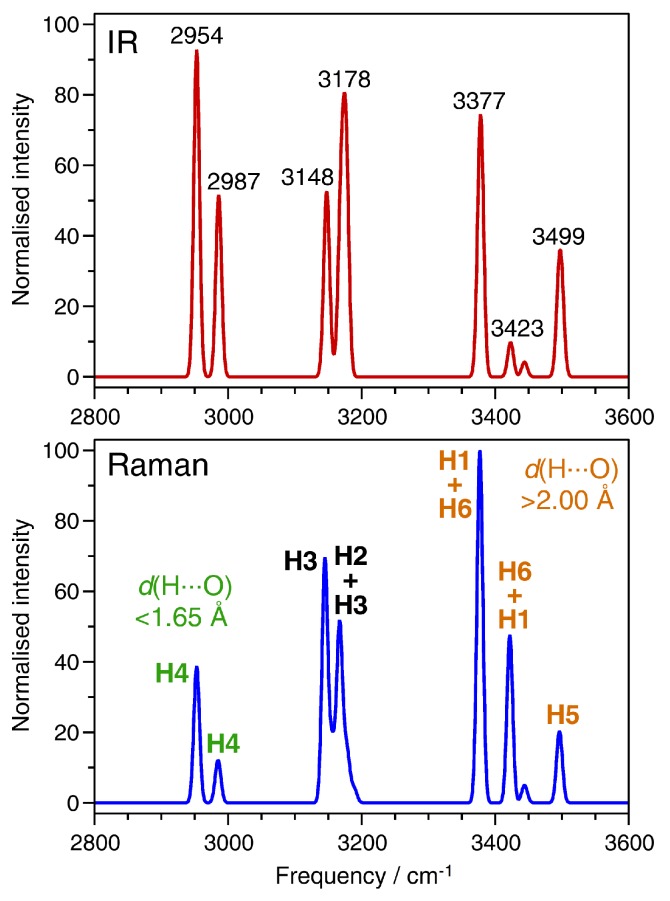
Computed IR (**top**) and Raman (**bottom**) spectra in the frequency range of O-H stretching vibrations. The IR spectrum includes selected frequency labels, whereas the Raman spectrum includes labels designating the primary atomic displacements associated with the modes.

The first part of this analysis deals with the O-H stretching region between ∼2900 and 3600 cm^−1^. The IR and Raman spectra calculated for this frequency region are shown in Figure 4. All bands are shifted towards lower frequencies (red-shifted) with respect to free water. This red-shift is a consequence of the elongation of the covalent O-H bonds caused by the involvement of the water molecules in hydrogen bonds [37]. The assignment of displacements of the non-equivalent hydrogen atoms to different modes is summarised in Table 4, together with the O-H and H···O distances for each H atom. Starting from the high-frequency end of the spectrum, the bands at ∼3500 cm^−1^ are predominantly associated with displacements of the H5 atom, which participates in the bifurcated hydrogen bond, having the shortest O-H bond length (i.e., least elongation compared to free water) and the longest H···O distances. The bands between ∼3380 and 3450 cm^−1^ are primarily due to vibrations of the H1 and H6 atoms. Although these hydrogen atoms belong to different types of water molecules (H6: H_2_O(pore), H1: Al-coordinated H_2_O(1)), they contribute to bands in the same frequency range because of similar hydrogen bond distances and, correspondingly, almost identical O-H bond lengths. The correlation between shorter hydrogen bonds (=more elongated O-H bonds) and a red-shift of the stretching frequency also holds for the other hydrogen atoms: H2 and H3 participate in hydrogen bonds with a length of roughly 1.8 Å, and they both contribute to bands between 3140 and 3190 cm^−1^, with some modes having essentially equal contribution from both hydrogen atoms. Finally, the hydrogen atom H4, which forms a very short hydrogen bond of ∼1.6 Å, gives rise to strongly red-shifted bands below 3000 cm^−1^. In their experimental study, Knops-Gerrits et al. assigned the two rather diffuse bands at lower frequencies to Al-coordinated water molecules, and the sharper, intense bands at the highest frequencies to water molecules in the channels (Figure 2 shows the experimental data) [7]. Although this assignment is largely confirmed by the calculations, we have to note that the hydrogen atom H1, despite being part of an Al-coordinated water molecule, contributes to high-frequency bands because it participates in a relatively long hydrogen bond.

**Figure 5:**
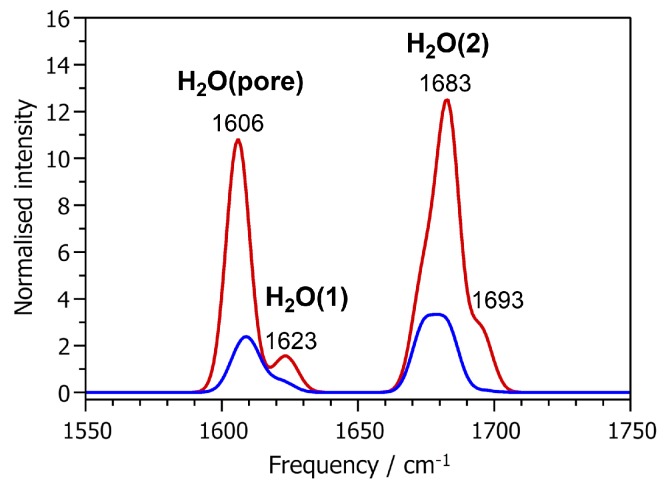
Computed IR (**red curve**) and Raman (**blue curve**) spectra in the frequency range of H-O-H bending vibrations.

In the frequency range of H-O-H bending vibrations, two main bands are visible in the IR and Raman spectra, shown in Figure 5. Both bands are comprised of contributions from several individual modes and are therefore relatively broad. The lower-frequency band extends from about 1600 to 1630 cm^−1^, and is thus close to the frequency of the H-O-H bending mode in a free water molecule (1607 cm^−1^ in PBE-TS calculations). The other band is blue-shifted with respect to free water, extending from about 1670 to 1700 cm^−1^. An analysis of the atomic displacements shows that the lower-frequency band arises from both H_2_O(pore) and H_2_O(1): The hydrogen atoms H5 and H6 of the H_2_O(pore) molecule contribute mainly to modes between 1606 and 1611 cm^−1^, whereas H1 and H2 contribute to modes between 1619 and 1625 cm^−1^. Consequently, the higher-frequency band is predominantly associated with displacements of the H3 and H4 atoms, which belong to the H_2_O(2) molecule. As shown in Table 5, there is no correlation between the frequency and the α(H-O-H) angle. The differences in the frequencies of the H-O-H bending modes are primarily related to the length (and thus, strength) of the hydrogen bonds in which the water molecules participate: The two strong hydrogen bonds of H_2_O(2) lead to an increase in energy (and hence frequency) of the H-O-H bending deformation, whereas the longer, weaker hydrogen bonds in which H_2_O(1) and H_2_O(pore) participate have only a modest effect. Taking together the observations made for O-H stretching and H-O-H bending modes, we can conclude that their frequencies are determined by the hydrogen bonding environment of the water molecules. Because the hydrogen bonding patterns may be different for non-equivalent Al-coordinated water molecules (as is the case for H_2_O(1) and H_2_O(2)), or rather similar for Al-coordinated water molecules and molecules adsorbed in the pores, the modes in this frequency range are no reliable indicator to distinguish between water molecules in different environments.

**Figure 6:**
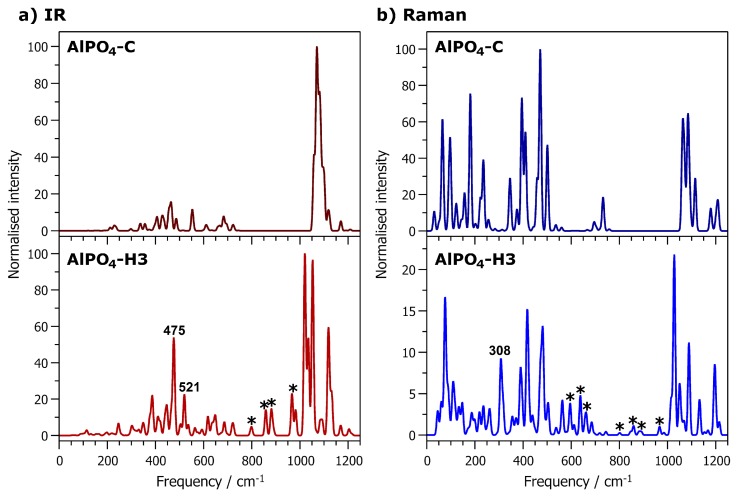
Computed IR (**left**) and Raman (**right**) spectra of AlPO_4_-C (**top)** and AlPO_4_-H3 (**bottom**). Frequency labels are included for modes that are discussed prominently in the text, whereas asterisks indicate modes that are mentioned summarily.

Finally, we turn our attention to the “framework modes”, i.e., all modes up to ∼1200 cm^−1^. The IR and Raman spectra of fully hydrated AlPO_4_-H3 and calcined AlPO_4_-C are shown in Figure 6. It is worth noting that the spectra display some common features, but also various differences, which are too numerous to discuss individually. Therefore, the present analysis focusses on a few selected bands that have significant intensity in the hydrated phase, but are absent or very weak in the calcined phase. One such band is found in the Raman spectrum at a frequency of 308 cm^−1^. It is associated primarily with displacements of the octahedrally coordinated Al2 atom and the attached oxygen atoms, i.e., deformations of the AlO_6_ octahedra. It is worth noting that an intense band at 326 cm^−1^ in the Raman spectrum of variscite was attributed to Al-O stretching vibrations [38]. Variscite is a hydrated aluminophosphate with composition AlPO_4_·2H_2_O that contains only octahedrally coordinated aluminium atoms. At frequencies around 475 cm^−1^, there are a few modes that give rise to a prominent band in the IR spectrum of AlPO_4_-H3 that has no direct equivalent in AlPO_4_-C. These modes are associated with a displacement of Al2 from the centre of the AlO_6_ octahedron, corresponding to a combination of Al-O bond stretching and O-Al-O angle bending. Another IR-active mode at 521 cm^−1^ is related to an off-centre displacement of the Al2 atom within the octahedron, but also has a strong contribution from the H_2_O(1) molecule.

Moving towards higher frequencies, there are several other bands in the IR and Raman spectra of AlPO_4_-H3 that have no counterpart in the spectra of AlPO_4_-C, e.g., Raman-active modes at 597 cm^−1^, 639 cm^−1^, 663 cm^−1^, as well as several IR- or (weakly) Raman-active modes between 800 and 1000 cm^−1^ (indicated by asterisks in Figure 6). An analysis of the displacement patterns shows that these modes are primarily associated with librations of the H_2_O molecules, sometimes coupled to internal deformations of the PO_4_ tetrahedra. This is in accordance with a previous IR and Raman spectroscopic analysis of variscite and metavariscite, where modes between ∼600 and 870 cm^−1^ were assigned to H_2_O librations [38]. Finally, although there are significant differences in the spectra above 1000 cm^−1^, all modes in this frequency range are due to P-O bond stretching vibrations, and therefore not directly related to the presence of AlO_6_ octahedra.

### 2.5. Displacement Parameters and Dynamic Behaviour

**Figure 7:**
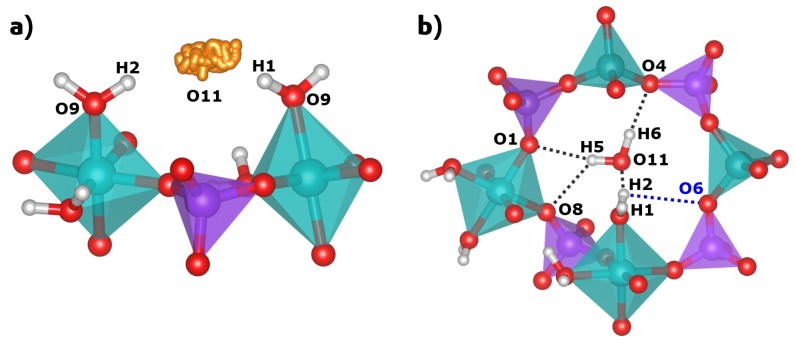
(**a**) Evolution of the position of one O11 atom (H_2_O(pore) molecule) during the 7.5 ps covered by the AIMD simulation. (**b**) Local environment of the H2 atom in the PBE-TS optimised structure. The contact to the O6 framework atom is shown in blue.

The root mean square displacements (RMSDs) obtained from the AIMD simulations are shown in Table 6. For the framework atoms, we find RMSDs in the range of 0.14 Å for aluminium and phosphorus atoms, and RMSDs of 0.18 to 0.23 Å for the bridging oxygen atoms. Unsurprisingly, the variations among the non-equivalent atoms of each group are not significantly larger than the typical standard deviations, in line with the expected rigidity of the framework. When looking at the three different water molecules, a much larger variation of the RMSDs is found: For the H_2_O(2) molecule, the RMSD of the O10 oxygen atom of 0.19 Å is similar to those of framework O atoms, indicating that this strongly bonded water molecule exhibits no significant mobility. The RMSDs of the two attached hydrogen atoms, H3 and H4, are only slightly larger, because the participation in strong and short hydrogen bonds limits the extent of displacements from their equilibrium positions. For the H_2_O(1) molecule, the RMSD of the O9 oxygen atom is only slightly larger than that of the O10 atom and of the framework oxygens. On the other hand, the RMSDs of the H1 and H2 atoms are much larger, amounting to 0.36 and 0.51 Å, respectively. While it seems plausible that these hydrogen atoms, which participate in longer hydrogen bonds than those of the H_2_O(2) molecule, undergo more pronounced oscillations, it has to be noted that the H2···O11 hydrogen bond in the PBE-TS equilibrium structure is much shorter than the H1···O11 bond. Therefore, one should expect a smaller RMSD for H2 compared to H1, contrary to the results of the AIMD calculations. We will revisit this point below, when discussing the radial distribution functions. Finally, the RMSDs for all three constituent atoms of the H_2_O(pore) molecule are in the range of ∼0.55 Å, much larger than for all other atoms except H2. Apparently, this water molecule is subject to pronounced oscillations, which is straightforwardly explained with the absence of an Al-O_water_ bond, leading to a significant freedom of movement along the channel. A visualisation of the positions visited by a single O11 atom over the course of the whole AIMD trajectory shows the large displacement of the H_2_O(pore) molecule (Figure 7a). The individual *x*, *y*, and *z* coordinates over the trajectory vary by 0.8/1.0/1.2 Å, corroborating that the most pronounced displacement occurs along the channel axis (*c*-axis). Given the large mobility of the H_2_O(pore) molecule found in the AIMD simulations, it is not surprising that the static equilibrium position of O11 obtained from the PBE-TS optimisations deviates from the experimentally observed position, which corresponds to the time-averaged position at room temperature.

Isostropic displacement parameters $U_{iso}$ can be obtained from electronic structure calculations via two different routes: On the one hand, they can be calculated from an AIMD trajectory, where they are directly related to the RMSDs. On the other hand, anisotropic displacement parameters can be routinely computed in the context of a lattice dynamical calculation, and the conversion into an equivalent isostropic value is straightforward. Table 6 lists both types of DFT-derived $U_{iso}$ values, dubbed as $U_{iso}$(AIMD) and $U_{iso}$(phonon), respectively, as well as experimental values [10]. If we compare the displacement parameters for all atoms except those belonging to the water molecules (i.e., including Al, P, and O1 to O8 atoms), a purely qualitative analysis indicates reasonably good agreement with experiment for both types of computed $U_{iso}$ values. A more quantitative assessment can be made by plotting the DFT-derived values as a function of their experimental counterparts. These two plots are shown in the Supporting Information (Figures S2 and S3). Here, we find excellent agreement between the $U_{iso}$(AIMD) values and experiment, with a correlation coefficient $R^{2}$ = 0.93 and a slope of the trendline of 0.98 (perfect agreement would result in a slope of 1). The predicted values obtained from the phonon calculation are in somewhat less good agreement, with a correlation coefficient of $R^{2}$ = 0.82 and a slope of 1.13, i.e., the calculations tend to overestimate $U_{iso}$. However, given the likely uncertainties of both experimental and calculated values, this agreement can still be considered satisfactory.

With regard to the water molecules, we observe a rather good agreement between both kinds of calculated $U_{iso}$ values and experiment for the oxygen atoms O9 and O10, which belong to the Al-coordinated water molecules. In contrast, the DFT-derived values for the hydrogen atoms are always much smaller than experimental values, which reach up to 0.20 Å. However, we have to note that the experimental values are likely to be inaccurate due to the difficulties in determining the hydrogen positions accurately (note especially that the O-H distances and H-O-H angles were fixed in the structure refinement, and that deviations in the real structure from these ideal values would translate into an increase of the displacement parameters). As such, we consider the experimental displacement parameters unreliable, and focus on a comparison of the different DFT-derived values. The $U_{iso}$ values obtained for the hydrogen atoms of the H_2_O(2) molecule differ appreciably among the two methods, but both methods agree on their relatively modest magnitude ($U_{iso}$ < 0.03 Å). For the H_2_O(1) molecule, the phonon calculation delivers increased $U_{iso}$ values, which are similar for both hydrogen atoms. As discussed above, a different picture arises when looking at the AIMD results, where the displacement parameter of the H2 atom is more than twice as large than that of H1. For the H_2_O(pore) molecule, the two computational methods give very different displacement parameters for all three atoms, with the $U_{iso}$(AIMD) values being a factor of 2 to 3 larger than those from the phonon calculations. The phonon calculations only consider harmonic oscillations around the equilibrium position, however, the significant displacements of the H_2_O(pore) molecule predicted by the AIMD calculations indicate that the harmonic approximation is no longer valid. As these effects are captured only by the molecular dynamics approach, we may consider the displacement parameters obtained with this method to be more reliable. Interestingly, the experimental $U_{iso}$ value of the O11 atom falls between the two computed values. However, the fact that the hydrogen positions of the H_2_O(pore) molecule could not be determined is likely to cause a rather large inaccuracy of this displacement parameter.

The radial distribution functions (RDFs) of the H···O hydrogen bonds obtained from the AIMD calculations are visualised in Figures S5–S7 of the Supporting Information. For the H_2_O(2) molecule, both the H3···O5 and the H4···O7 RDF exhibit one sharp first maximum centered at about 1.73 Å and 1.60 Å, respectively, in excellent agreement with the equilibrium H···O distances obtained in the PBE-TS structure optimisation (Table 2). Looking at the H_2_O(1) molecule, the H1···O11 RDF has a somewhat broader maximum at a distance of ∼1.73 Å. This is significantly shorter than the PBE-TS equilibrium distance of 2.06 Å, a difference that can be attributed to the mobility of the H_2_O(pore) molecule, which is not accounted for in the static calculation. For the H2 atom, there is one maximum in the H2···O11 centered at ∼1.84 Å, which agrees well with the PBE-TS distance. Additionally, a pronounced tail is observed towards longer distances, and a calculation of the RDF between H2 and different framework oxygen atoms reveals that there are also short contacts to the framework oxygen atom O6 occurring over the course of the AIMD simulation. The first maximum in the H2···O6 RDF is close to ∼2 Å, well below the PBE-TS equilibrium distance of 2.88 Å. This indicates that the hydrogen bonding situation of the H2 atom is dynamic, rather than static, with H2···O11 and H2···O6 hydrogen bonds forming and breaking over time (the environment of the H2 atom including both O11 and O6 is shown in Figure 7b). The dynamic H-bonding of H2 also explains why its $U_{iso}$(AIMD) value is so much larger than $U_{iso}$(phonon). For the H_2_O(pore) molecule, the RDFs for the H6···O4 hydrogen bond and the bifurcated hydrogen bond (H5···O1, H5···O8) all exhibit a single maximum centered at the respective equilibrium distances. These maxima are relatively broad due to the high mobility of the H_2_O(pore) molecule.The RDFs of intramolecular O-H bonds and of Al-O bonds are also included in the Supporting Information (Figures S4 and S8). In all cases, the position of the first maximum in the RDF hardly deviates from the equilibrium distance in the PBE-TS optimised structure. For both groups of bonds, the maxima become broader with increasing equilibrium bond length. This effect is particularly pronounced for the Al-O bonds, where bonds to water molecules are somewhat more flexible than bonds to framework oxygen atoms.

## 3. Discussion

The comparative DFT calculations for partially hydrated phase clearly indicate that such phases are not stable, regardless of which water molecule is removed. The predictions are in line with the reported dehydration behaviour [20], and also agree with earlier calculations on CHA-type AlPO-34 [14]. Apparently, even in the small-pore APC-type structure, Al-coordinated H_2_O molecules are stabilised only through hydrogen bonds to other water molecules adsorbed in the channels. Since the APC-type AlPO is one of the porous aluminophosphates with the smallest pores, we may expect that this finding can be generalised to all porous AlPOs, regardless of the pore size. The situation is different for dense hydrated aluminophosphates like variscite and metavariscite, and possibly also for porous AlPOs where water molecules are able to adsorb in a bridging position, simultaneously coordinating to two framework Al atoms. Such a scenario has been described for AlPO-14, where the water desorption proceeds in two steps, and where the second step was attributed to the desorption of water molecules bridging between two Al sites [39].

A prediction of the IR and Raman spectra of AlPO_4_-H3 showed that the three non-equivalent water molecules give rise to different bands in the frequency range of O-H bond stretching. An experimental Raman spectrum has been reported for this frequency range, and the excellent agreement of frequencies (apart from a constant scaling factor) and intensities gives confidence in the computational predictions. The frequency shift with respect to a free water molecule is determined by the hydrogen bonding pattern, so information about the length/strength of the hydrogen bonds in which the adsorbed water molecules participate can be inferred from these modes. Using such complementary information from vibrational spectroscopy could help to make reasonable guesses of the hydrogen positions in cases where only the positions of the water oxygen atoms can be determined (e.g., from PXRD data). While modes in the O-H stretching frequency range cannot be used as “fingerprints” to distinguish Al-coordinated water molecules from molecules adsorbed in the pores of AlPO_4_-H3, a few “framework modes” in the frequency range below ∼1200 cm^−1^ that are predominantly related to deformations of AlO_6_ octahedra were identified. In particular, a Raman-active mode at 308 cm^−1^ that seems to have a counterpart in variscite and a group of IR-active modes at ∼475 cm^−1^ could be modes that are indicative for the presence of octahedrally coordinated Al. To establish whether these modes are really useful as “fingerprints”, experimental work on hydrated aluminophosphates should be performed, focussing on systems for which some information about the presence of Al-coordinated water molecules is available (e.g., from diffraction and/or NMR spectroscopy).

Finally, DFT-based AIMD calculations were used to analyse the dynamic behaviour of the water molecules. Given its different bonding environment, it is not surprising that the H_2_O(pore) molecule exhibits a higher mobility than the Al-coordinated water molecules. The large mobility may also serve to explain the deviations between the position of this molecule in the DFT-optimised structure and the experimentally observed position. The Al-coordinated H_2_O(1) molecule is capable of dynamically changing its H-bonding pattern, and its behaviour is related to the mobility of H_2_O(pore): If the H_2_O(pore) molecule moves so far away that the H2···O11 H-bond is elongated beyond a certain extent, the O9-H2 bond reorients to form a hydrogen bond to the framework oxygen atom O6. While the dynamic behaviour of this water molecule could not be deduced from the experimental crystal structure, it has to be noted that previous AIMD studies of other hydrated AlPOs and related systems also highlighted the importance of dynamic hydrogen bonding [40]: For example, thermal oscillations of the water molecules adsorbed in the channels of the large-pore AlPO VPI-5 cause a continuous breaking and reformation of hydrogen bonds without there being any appreciable diffusion [41]. Another interesting observation is the good agreement of $U_{iso}$ values calculated using either AIMD or phonon calculations with experimental values. This indicates that $U_{iso}$ parameters taken from calculations could be used as realistic starting values in structure refinements, which might be especially useful in PXRD studies.

## 4. Materials and Methods

### 4.1. Computational Details

Structure optimisations and calculations of vibrational spectra were performed with the CASTEP code (version 17.2), which uses plane waves and pseudopotentials, and which can impose constraints consistent with the space group symmetry [42]. The core electrons were represented using norm-conserving pseudopotentials taken from the database of Bennett and Rappe (http://www.sas.upenn.edu/rappegroup/research/pseudo-potential-gga.html) [43]. An energy cutoff of 1000 eV was used. The calculations were carried out using the PBE exchange-correlation functional [22] in conjunction with the dispersion correction devised by Tkatchenko and Scheffler [23], as the PBE-TS method was found to give fairly accurate results in previous studies of aluminophosphates [24,33,44,45,46]. Due to the size of the unit cell of AlPO_4_-H3/AlPO_4_-C, only the Γ point was used to sample the Brillouin zone. Calculations of the vibrational properties, including IR and Raman intensities, used the density functional perturbation theory formalism as implemented in CASTEP [47,48,49,50,51]. To determine the interaction strength between framework and adsorbed water molecules, both the interaction energy $\Delta E_{int}$ and the adsorption enthalpy $\Delta H_{ads}$ for *T* = 298 K were calculated for fully hydrated AlPO_4_-H3 and several partially hydrated models. The calculations of the interaction energy per water molecule made use of the DFT energies $E_{\mathrm{PBE}-\mathrm{TS}}$ obtained from the structure optimisations:

(1) $$\Delta E_{int}=\left(E_{\mathrm{PBE}-\mathrm{TS}}\left({\mathrm{APC}}_{\mathrm{hydr}}\right)-E_{\mathrm{PBE}-\mathrm{TS}}\left({\mathrm{APC}}_{\mathrm{dehydr}}\right)-n\cdot E_{\mathrm{PBE}-\mathrm{TS}}\left(H2O\right)\right)/n$$

Here, APC_hydr_ and APC_dehydr_ refer to a hydrated model and to dehydrated AlPO_4_-C, respectively. The energy for a single water molecule was obtained from a calculation for a water molecule in a box with an edge length of 15 Å. To calculate the adsorption enthalpy $\Delta H_{ads}$, the corresponding zero-point vibrational energy and vibrational contributions at *T* = 298 K, obtained from the lattice dynamical calculations, were added to each term on the right-hand side of the above equation (it has to be noted that these contributions will be of limited accuracy because only the Γ point was sampled in the calculations). The lattice dynamical calculations also delivered the anisotropic displacement parameters $U_{ij,x}$ for each non-equivalent atom *x*. The isotropic displacement parameters $U_{iso}$ were calculated from these values using the well-established approximation $U_{iso,x}=\left(U_{11,x}+U_{22,x}+U_{33,x}\right)/3$ [52].

DFT-based ab initio molecular dynamics (AIMD) calculations were carried out for AlPO_4_-H3, using a 1×2×2 supercell of the crystallographic unit cell. The AIMD simulations were performed using the CP2K code (version 2.6.2, installed on the HLRN supercomputer “Konrad”), which employs a hybrid Gaussian and plane wave scheme [53,54]. Due to unavailability of the PBE-TS functional in CP2K, the PBE functional was used in conjunction with the Grimme-type D3 dispersion correction [22,55]. Furthermore, these calculations used a plane wave energy cutoff of 500 Ry, Goedecker-Teter-Hutter pseudopotentials devised by Krack [56], and a DZVP-MOLOPT basis set. Only the Γ point was used to sample the first Brillouin zone. The AIMD simulations were performed in the canonical (NVT) ensemble for a temperature of 298 K, using a Nosé-Hoover thermostat, a timestep of 0.5 fs and a time constant of 50 fs. The full MD trajectory consisted of 20,000 steps (10 ps), of which the first 5000 steps were used for equilibration. The analysis of the “production” part of the trajectory was carried out with the VMD software, version 1.9.3 [57]. From the root mean square displacements (RMSDs) calculated with VMD, the isotropic displacement parameters were calculated as $U_{iso,x}=\frac{1}{3}{\left({\mathrm{RMSD}}_{x}\right)}^{2}$ [52].

### 4.2. Structure Models

The starting structure for the DFT optimisation of AlPO_4_-H3 was taken from the work of Pluth and Smith [10], the only modification being the addition of two hydrogen atoms (H5 and H6) to the O11 atom of the H_2_O(pore) molecule. These atoms were added in a way that the angles between the H1···O11 and H2···O11 hydrogen bonds and the O11-H5 and O11-H6 bonds correspond approximately to the tetrahedral angle. Partially hydrated models were constructed by starting from the PBE-TS optimised structure of AlPO_4_-H3 and removing either one or two water molecules, leading to a total of six different partially hydrated models. The structure of fully dehydrated AlPO_4_-C was also optimised. Here, the initial structure was taken from the PXRD study by Keller, Meier, and Kirchner [21].

## 5. Conclusions

The calculations reported in this work have delivered insights into structure, energetics, vibrational properties, and dynamic behaviour of AlPO_4_-H3. It has to be reiterated that this system is only of limited relevance to applications due to its small pores and the resulting low water uptake, however, the methods applied here could easily be transferred to other AlPOs that are currently considered as promising adsorbents for thermal energy storage (e.g., AlPO-34, AlPO-18, LTA-type AlPO). A key issue for these larger-pore systems is the construction of starting models of the hydrated structure. Even if the crystal structure of the hydrated form has been reported (as is the case for AlPO-34 and AlPO-18 [8,9,12]), the H-bond network will typically be less ordered than in AlPO_4_-H3, meaning that the setup of the starting structure would need to consider several possible arrangements. Another potential problem is a lack of long-range ordering of the Al-coordinated water molecules, which could be bonded to different framework Al atoms in different unit cells. In principle, it is possible to predict the most likely positions of the Al-coordinated water molecules by performing calculations for several partially hydrated models, as done previously for AlPO-11 [58]. However, it has to be kept in mind that such partially hydrated phases are thermodynamically metastable, and that the interaction with “pore” water molecules will contribute to the stabilisation of an Al-coordinated water molecule in a given position.

An analysis of the vibrational spectra in the O-H stretching frequency range can provide insights into the relative importance of shorter/stronger and longer/weaker hydrogen bonds in hydrated AlPOs; however, a direct assignment of an observed band to one particular water molecule in the structure will normally not be possible. Even in AlPO_4_-H3, where the water molecules are highly ordered, some bands stem from displacements of two H atoms belonging to different molecules, and such overlapping contributions will become more pronounced in more complex structures. In contrast, the “framework modes” attributed to Al-coordinated water molecules should remain largely unaffected, and they could become a useful indicator for the presence of Al-coordinated water molecules.

Finally, the AIMD calculations show that dynamic effects are important even in this well-ordered structure. The inherent limitations of a purely static approach are especially well visible when trying to reconcile the calculated equilibrium position of H_2_O(pore) with the experimentally observed position. Given the ever-increasing computing power, AIMD calculations should become more and more routinely feasible in the future, and the field of porous materials provides a wide range of potential applications besides hydrated zeotypes, such as the dynamic behaviour of gases like CO_2_ in adsorbent materials, the dynamics of extra-framework cations in charged-framework zeolites, and the behaviour of organic structure-directing agents confined to the pores of as-synthesised zeotypes [40].

## Figures and Tables

**Table 1:** Comparison of experimental and DFT-optimised lattice parameters and T-O bond distances for AlPO_4_-H3 and AlPO_4_-C. For T-O bonds in AlPO_4_-H3, SRB-corrected values of the experimental bond lengths are given in brackets. *d_aver_* represents averages over several bonds, O_fw_ designates framework oxygen atoms.

	AlPO_4_-H3		AlPO_4_-C	
	sc-XRD [10]	DFT	PXRD [21]	DFT
**Unit cell parameters**				
*a*/Å	19.353	19.498	19.816	20.392
*b*/Å	9.727	9.751	10.047	9.648
*c*/Å	9.762	9.795	8.935	8.825
*V*/Å^3^	1837.7	1862.3	1778.9	1736.3
**T-O bond lengths**				
*d_aver_*(P-O_fw_)/Å	1.524 (1.530)	1.540	1.523	1.539
*d_aver_*(Al1-O_fw_)/Å	1.733 (1.739)	1.758	1.730	1.757
*d_aver_*(Al2-O_fw_)/Å	1.847 (1.851)	1.873	-	-
*d*(Al2-O9)/Å	1.967 (1.977)	1.981	-	-
*d*(Al2-O10)/Å	1.951 (1.956)	1.965	-	-

**Table 2:** Hydrogen bond distances and angles in AlPO_4_-H3.

	sc-XRD	DFT
**H_2_O(1)**		
*d*(O9···O11(A))	2.893 Å	3.020 Å
*d*(H1···O11(A))	2.070 Å	2.055 Å
α(O9-H1···O11(A))	162.8${}^{\circ }$	167.7${}^{\circ }$
*d*(O9···O11(B))	3.011 Å	2.791 Å
*d*(H2···O11(B))	2.183 Å	1.821 Å
α(O9-H2···O11(B))	164.5${}^{\circ }$	165.1${}^{\circ }$
**H_2_O(2)**		
*d*(O10···O5)	2.752 Å	2.722 Å
*d*(H3···O5)	1.990 Å	1.752 Å
α(O10-H3···O5)	148.7${}^{\circ }$	163.9${}^{\circ }$
*d*(O10···O7)	2.603 Å	2.578 Å
*d*(H4···O7)	1.790 Å	1.603 Å
α(O10-H4···O7)	159.4${}^{\circ }$	162.2${}^{\circ }$
**H_2_O(pore)**		
*d*(O11···O8)	3.047 Å	3.061 Å
*d*(H5···O8)	-	2.349 Å
α(O11-H5···O8)	-	129.1${}^{\circ }$
*d*(O11···O1)	3.213 Å	3.267 Å
*d*(H5···O1)	-	2.369 Å
α(O11-H5···O1)	-	152.4${}^{\circ }$
*d*(O11···O4)	3.013 Å	2.995 Å
*d*(H6···O4)	-	2.027 Å
α(O11-H6···O4)	-	169.6${}^{\circ }$

**Table 3:** Calculated interaction energies and adsorption enthalpies for fully hydrated AlPO_4_-H3 and derived partially hydrated phases. All values are given per H_2_O molecule.

	H_2_O(1)	H_2_O(2)	H_2_O(pore)	$\Delta E_{\mathrm{int}}$/kJ mol^−1^	$\Delta H_{\mathrm{ads}}$/kJ mol^−1^
**Fully hydrated**	8	8	8	−81.8	−67.3
**2/3 hydrated**	8	8	0	−76.7	−62.5
	8	0	8	−70.6	−56.0
	0	8	8	−76.6	−62.8
**1/3 hydrated**	8	0	0	−44.8	−30.6
	0	8	0	−71.5	−57.7
	0	0	8	−67.8	−53.3

**Table 4:** Lengths of covalent O-H bonds and hydrogen bonds in AlPO_4_-H3 and frequencies of associated O-H stretching modes. PBE-TS values of an isolated water molecule are given for comparison.

	*d*(O-H)/Å	*d*(H···O)/Å	Mode Frequencies/cm^−1^
**H_2_O molecule**	0.9668	-	3584 (symmetric stretching)
			3699 (asymmetric stretching)
**AlPO_4_-H3**			
**H1**	0.9799	2.055	3377 to 3379 (secondary: ∼3445)
**H2**	0.9921	1.821	3168 to 3189
**H3**	0.9949	1.752	3145 to 3148 and 3178 to 3189
**H4**	1.0047	1.603	2954 to 2987
**H5**	0.9778	2.349/2.369	3496 to 3499
**H6**	0.9790	2.027	3423 to 3445 (secondary: ∼3375)

**Table 5:** Equilibrium H-O-H angles in AlPO_4_-H3 and frequencies of associated H-O-H bending modes. PBE-TS values of an isolated water molecule are given for comparison.

	α(H-O-H)/${}^{\circ }$	Mode Frequencies/cm^−1^
**H_2_O molecule**	104.21	1607
**AlPO_4_-H3**		
**H_2_O(1)**	112.39	1619 to 1625
**H_2_O(2)**	102.90	1674 to 1696
**H_2_O(pore)**	105.04	1606 to 1611

**Table 6:** Root mean square displacements and isotropic discplacement parameters $U_{iso}$ obtained from AIMD calculations as well as $U_{iso}$ obtained from phonon calculations. Experimental values are given for comparison [10].

	RMSD(AIMD)/Å	$U_{\mathrm{iso}}$(AIMD)/Å^2^	$U_{\mathrm{iso}}$(phonon)/Å^2^	$U_{\mathrm{iso}}$(sc-XRD)/Å^2^
**Framework**				
**Al1**	0.143 ± 0.015	0.0068	0.0079	0.0089
**Al2**	0.135 ± 0.015	0.0061	0.0069	0.0079
**P1**	0.142 ± 0.014	0.0067	0.0072	0.0084
**P2**	0.133 ± 0.015	0.0059	0.0070	0.0078
**O1**	0.211 ± 0.017	0.0148	0.0154	0.0146
**O2**	0.227 ± 0.020	0.0172	0.0193	0.0174
**O3**	0.228 ± 0.021	0.0173	0.0230	0.0163
**O4**	0.198 ± 0.017	0.0131	0.0140	0.0134
**O5**	0.183 ± 0.019	0.0112	0.0152	0.0114
**O6**	0.222 ± 0.016	0.0164	0.0190	0.0160
**O7**	0.203 ± 0.018	0.0137	0.0126	0.0133
**O8**	0.178 ± 0.018	0.0106	0.0148	0.0112
**H_2_O(1)**				
**O9**	0.244 ± 0.020	0.0198	0.0226	0.022
**H1**	0.357 ± 0.030	0.0425	0.0397	0.20
**H2**	0.506 ± 0.051	0.0853	0.0365	0.15
**H_2_O(2)**				
**O10**	0.188 ± 0.016	0.0118	0.0121	0.0155
**H3**	0.242 ± 0.022	0.0195	0.0278	0.066
**H4**	0.231 ± 0.022	0.0178	0.0242	0.054
**H_2_O(pore)**				
**O11**	0.552 ± 0.065	0.1016	0.0311	0.0568
**H5**	0.590 ± 0.075	0.1160	0.0602	-
**H6**	0.556 ± 0.063	0.1030	0.0465	-

## Supplementary Materials

The supplementary materials are available online. Table S1: Table of individual T-O distances, Table S2 and Figure S1: Structural parameters of partially hydrated phases, Tables S3 and S4, Figures S2 and S3: Displacement parameters, Figures S4 to S8: Radial distribution functions; other material: CIF files of DFT-optimised AlPO_4_-H3 and AlPO_4_-C; CASTEP *.phonon files of AlPO_4_-H3 and AlPO_4_-C.
