# Supplementary material for: First-Principles Study of AlPO4-H3, a Hydrated Aluminophosphate Zeotype Containing Two Different Types of Adsorbed Water Molecules
Source: Molecules. 2019 Mar 6;24(5):922. doi: 10.3390/molecules24050922 (PMC6429327; doi:10.3390/molecules24050922)
Supplement: Supplementary file 1 [file molecules-24-00922-s001.zip › Fischer_Molecules_2019_SupportingInformation.pdf]

First-principles study of $AlPO_4$-H3, a hydrated aluminophosphate zeotype containing two different types of adsorbed water molecules

SUPPORTING INFORMATION

Michael Fischer[1,2]

1 Crystallography Group, Department of Geosciences, University of Bremen, Klagenfurter Straße 2-4, 28359 Bremen, Germany

2 MAPEX Center for Materials and Processes, University of Bremen, 28359 Bremen, Germany

michael.fischer@uni-bremen.de

Individual T-O bond distances

Table S1: Table of experimental and DFT-optimised T-O bond distances in $AlPO_4$-H3 and $AlPO_4$-C. All bond lengths in Å.

	**$AlPO_4$-H3**		**$AlPO_4$-C**	
	**sc-XRD** [1]	**DFT**	**PXRD** [2]	**DFT**
**Al1-O2**	1.735	1.763	1.682	1.751
**Al1-O3**	1.724	1.749	1.744	1.758
**Al1-O4**	1.743	1.772	1.665	1.761
**Al1-O6**	1.729	1.749	1.773	1.762
**Al2-O1**	1.851	1.887	1.789	1.761
**Al2-O5**	1.859	1.880	1.771	1.756
**Al2-O7**	1.842	1.865	1.700	1.762
**Al2-O8**	1.835	1.859	1.719	1.742
**Al2-O9**	1.967	1.981	-	-
**Al2-O10**	1.951	1.965	-	-
**P1-O1**	1.501	1.516	1.492	1.540
**P1-O2**	1.531	1.547	1.590	1.544
**P1-O3**	1.529	1.543	1.475	1.534
**P1-O4**	1.538	1.558	1.551	1.540
**P2-O5**	1.525	1.543	1.495	1.535
**P2-O6**	1.535	1.552	1.568	1.544
**P2-O7**	1.527	1.546	1.493	1.539
**P2-O8**	1.506	1.515	1.517	1.532

Partially hydrated phases

Table S2: Lattice parameters of DFT-optimised partially hydrated APC phases.

	$H_2O(1)$	$H_2O(2)$	$H_2O(pore)$	***a*** / Å	***b*** / Å	***c*** / Å	***V*** / Å^3
**$AlPO_4$-H3**	8	8	8	19.498	9.751	9.795	1862.3
**2/3 hydrated**	8	8	0	19.819	9.309	9.942	1834.3
	8	0	8	19.598	10.193	8.973	1792.3
	0	8	8	15.723	9.679	9.681	1473.3
**1/3 hydrated**	8	0	0	19.655	9.768	9.781	1877.8
	0	8	0	16.653	10.101	9.627	1619.3
	0	0	8	21.415	9.380	8.328	1672.8
**$AlPO_4$-C**	0	0	0	20.392	9.648	8.825	1763.3

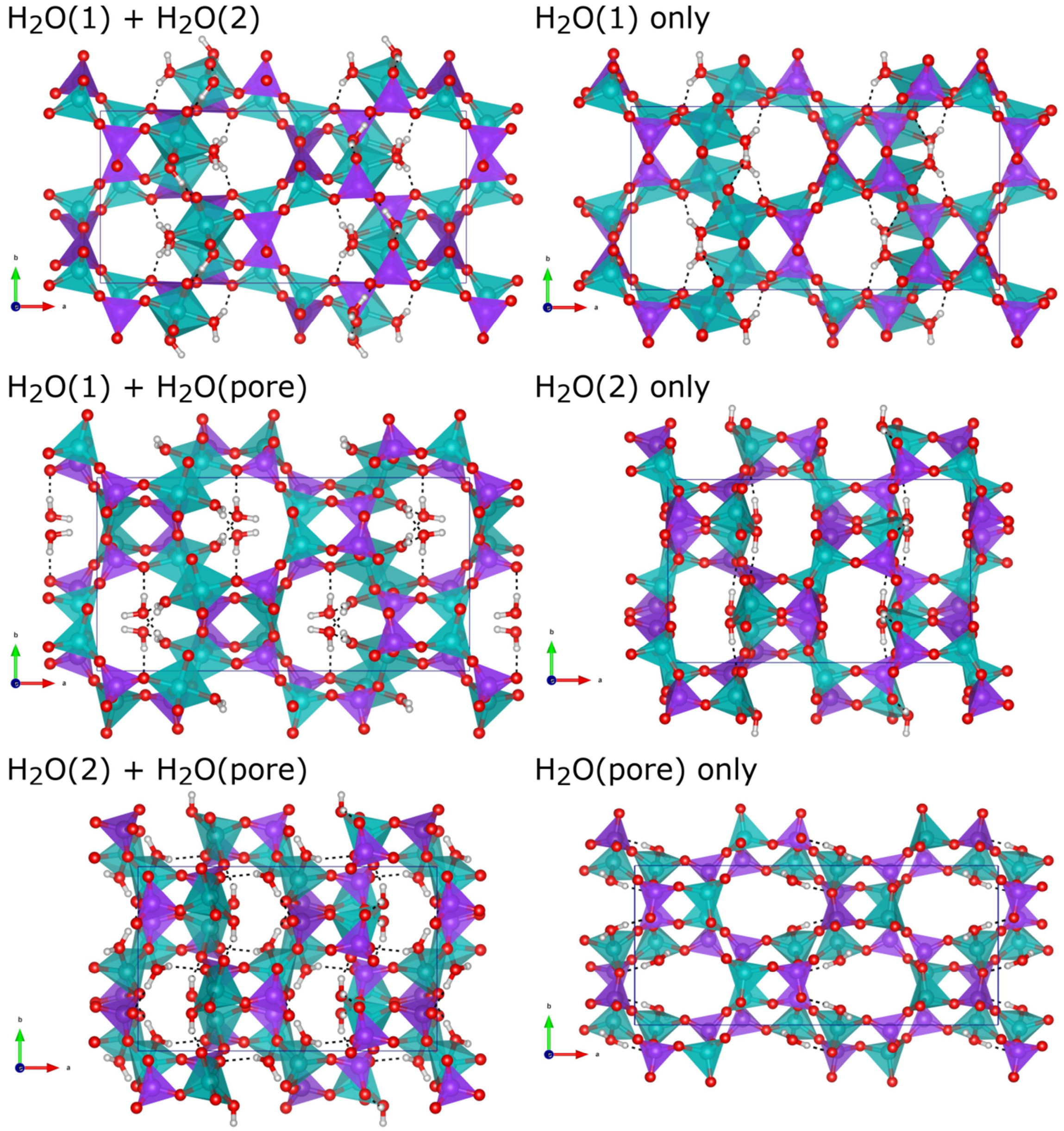

Figure S1: Visualisation of DFT-optimised partially hydrated APC phases.

Displacement parameters

Table S3: Anisotropic displacement parameters U_{ij} of $AlPO_4$-H3 as obtained from phonon calculations. The U_{iso}(phonon) values calculated from the anisotropic values are also included. All values in $Å^2$.

	U_{11}	U_{22}	U_{33}	U_{23}	U_{31}	U_{12}	U_{iso}
Al1	0.0068	0.0112	0.0058	0.0003	0.0002	0.0038	0.0079
Al2	0.0066	0.0085	0.0057	0.0006	0.0005	0.0024	0.0069
P1	0.0050	0.0102	0.0064	0.0015	-0.0002	-0.0026	0.0072
P2	0.0081	0.0077	0.0052	-0.0001	-0.0006	-0.0043	0.0070
O1	0.0069	0.0136	0.0257	-0.0017	0.0018	0.0002	0.0154
O2	0.0277	0.0233	0.0069	-0.0042	0.0019	-0.0037	0.0193
O3	0.0211	0.0282	0.0197	0.0012	-0.0036	-0.0201	0.0230
O4	0.0149	0.0161	0.0110	0.0053	0.0021	0.0050	0.0140
O5	0.0283	0.0121	0.0052	-0.0006	0.0008	-0.0099	0.0152
O6	0.0105	0.0157	0.0309	-0.0051	0.0003	-0.0008	0.0190
O7	0.0209	0.0093	0.0076	0.0021	-0.0012	-0.0025	0.0126
O8	0.0176	0.0167	0.0102	-0.0029	0.0036	0.0099	0.0148
O9	0.0093	0.0398	0.0188	0.0020	-0.0014	-0.0021	0.0226
H1	0.0231	0.0613	0.0345	0.0067	-0.0094	0.0018	0.0397
H2	0.0208	0.0571	0.0315	0.0009	0.0054	-0.0060	0.0365
O10	0.0177	0.0092	0.0094	-0.0012	-0.0004	-0.0007	0.0121
H3	0.0386	0.0193	0.0256	0.0012	0.0010	-0.0091	0.0278
H4	0.0354	0.0227	0.0146	-0.0003	0.0019	-0.0035	0.0242
O11	0.0168	0.0359	0.0406	0.0012	0.0021	-0.0002	0.0311
H5	0.0704	0.0448	0.0655	0.0052	-0.0106	-0.0195	0.0602
H6	0.0215	0.0607	0.0572	-0.0067	-0.0022	0.0040	0.0465

Table S4: Anisotropic displacement parameters U_{ij} of $AlPO_4$-C as obtained from phonon calculations. The U_{iso}(phonon) values calculated from the anisotropic values are also included. All values in $Å^2$.

	U_{11}	U_{22}	U_{33}	U_{23}	U_{31}	U_{12}	U_{iso}
Al1	0.0069	0.0109	0.0082	0.0025	0.0019	0.0042	0.0086
Al2	0.0069	0.0060	0.0101	0.0000	0.0017	0.0017	0.0077
P1	0.0066	0.0088	0.0063	-0.0002	0.0007	-0.0035	0.0072
P2	0.0064	0.0063	0.0102	0.0011	-0.0015	-0.0023	0.0076
O1	0.0078	0.0173	0.0266	0.0030	-0.0004	-0.0010	0.0172
O2	0.0237	0.0182	0.0087	0.0054	0.0017	0.0035	0.0169
O3	0.0146	0.0174	0.0109	-0.0023	0.0028	-0.0116	0.0143
O4	0.0199	0.0123	0.0100	0.0030	0.0009	0.0001	0.0141
O5	0.0206	0.0164	0.0126	0.0058	0.0010	-0.0007	0.0165
O6	0.0081	0.0222	0.0296	-0.0028	0.0012	0.0013	0.0200
O7	0.0241	0.0070	0.0149	0.0027	0.0002	-0.0007	0.0154
O8	0.0215	0.0154	0.0190	0.0016	0.0091	0.0119	0.0186

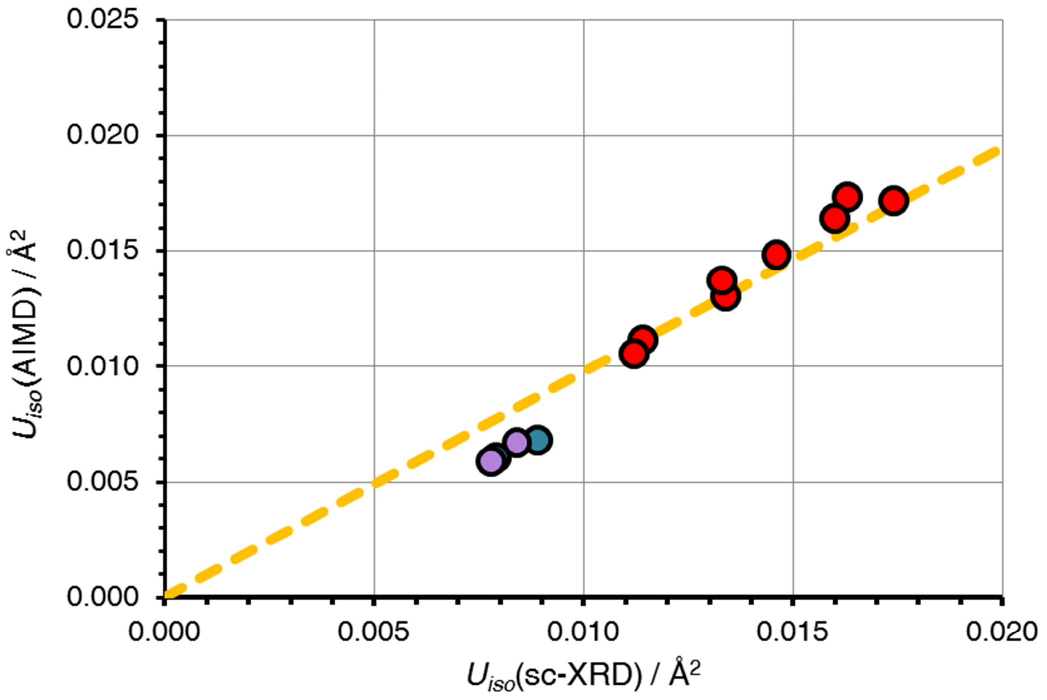

Figure S2: Plot of isotropic displacement parameters U_{iso} obtained from AIMD calculations as a function of the corresponding experimental values [1]. Only framework atoms are included (Al = cyan, P = purple, O1 to O8 = red). The yellow line shows a linear correlation with intercept 0.978 (R^2 = 0.930).

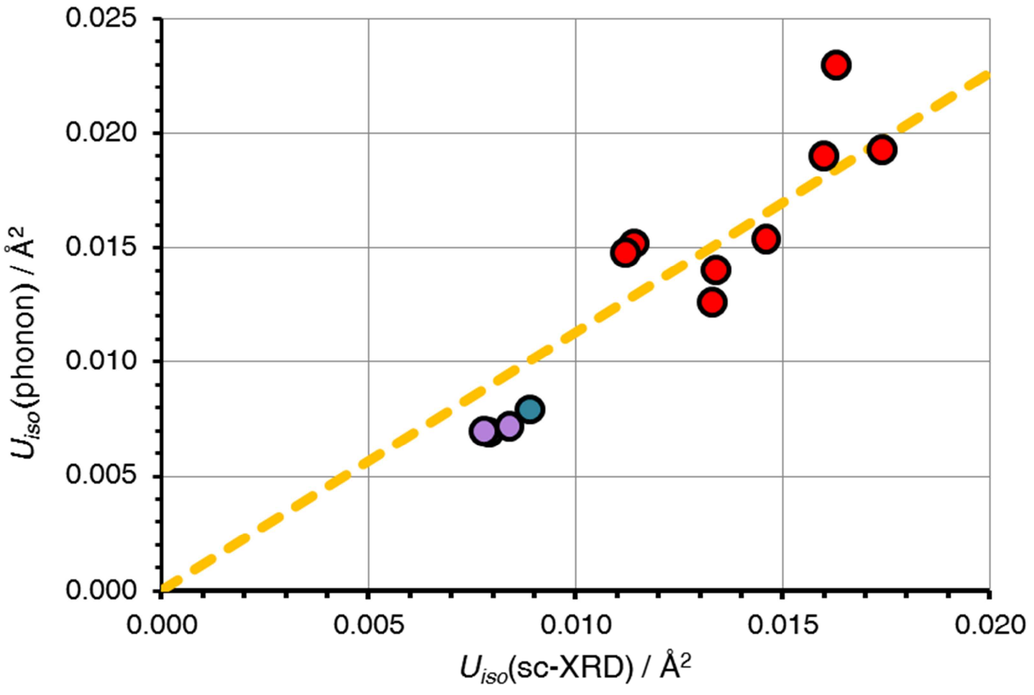

Figure S3: Plot of isotropic displacement parameters U_{iso} obtained from phonon calculations as a function of the corresponding experimental values [1]. Only framework atoms are included (Al = cyan, P = purple, O1 to O8 = red). The yellow line shows a linear correlation with intercept 1.131 (R^2 = 0.821).

Radial distribution functions

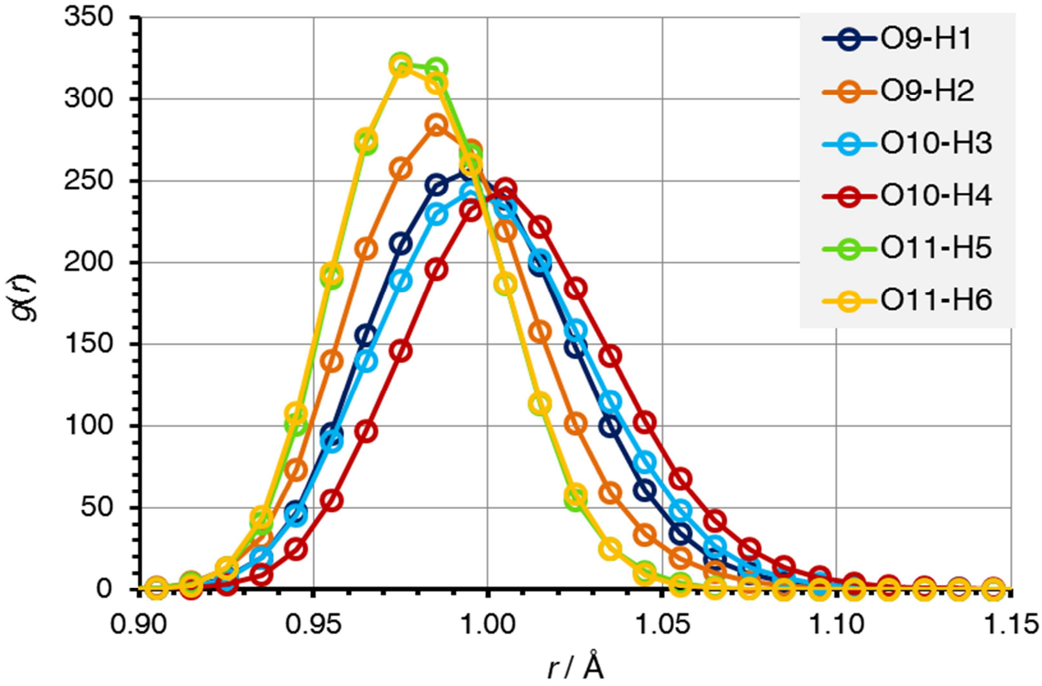

Figure S4: O-H radial distribution functions for the three water molecules.

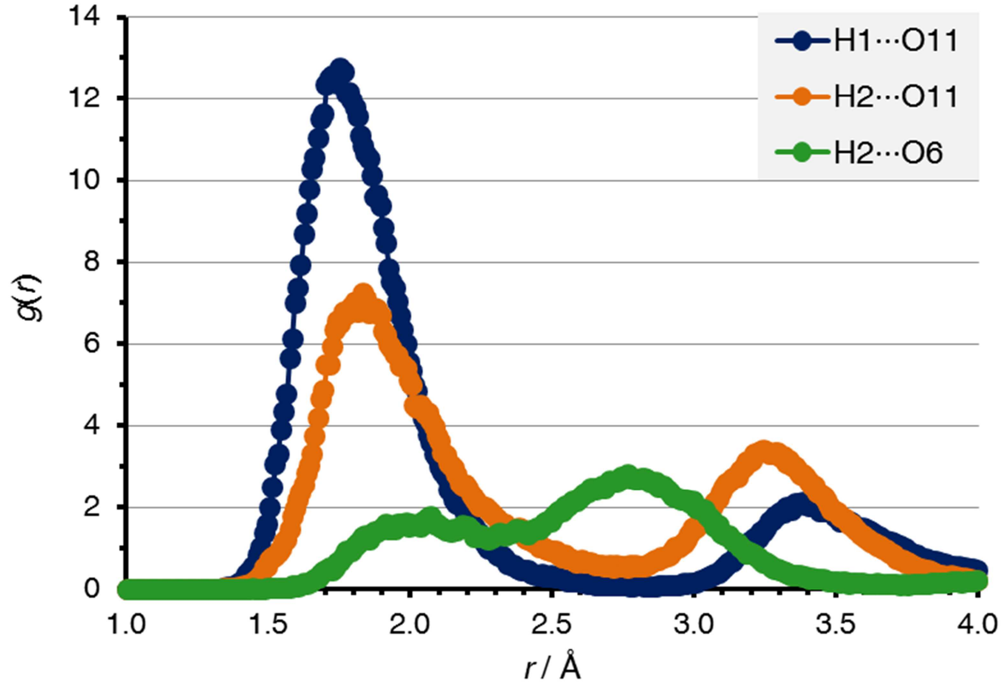

Figure S5: H···O radial distribution functions for the $H_2O(1)$ molecule.

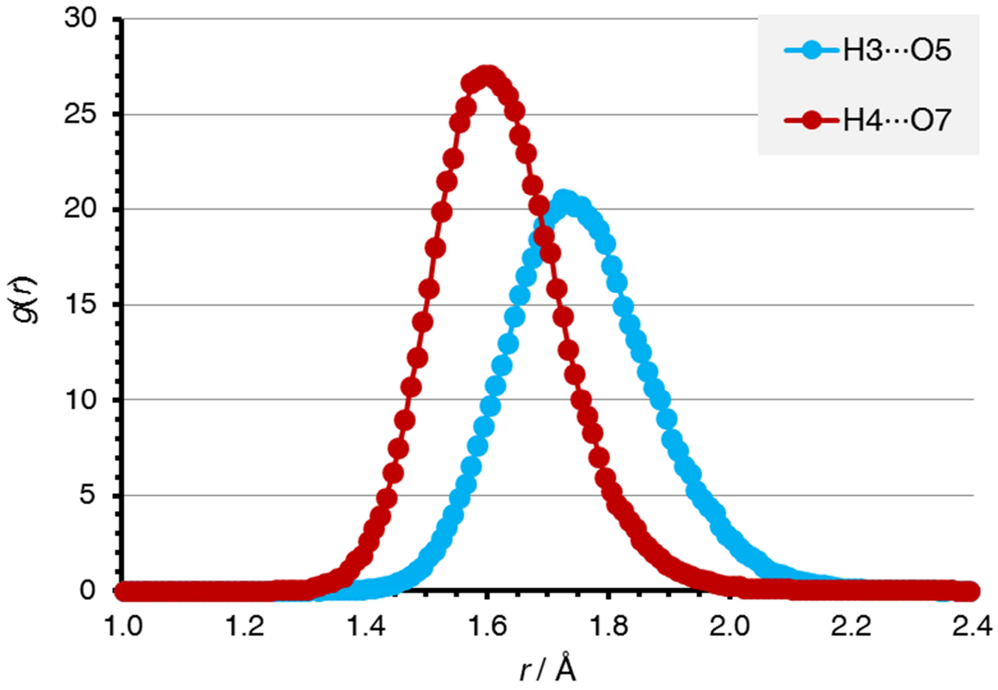

Figure S6: H···O radial distribution functions for the $H_2O(2)$ molecule.

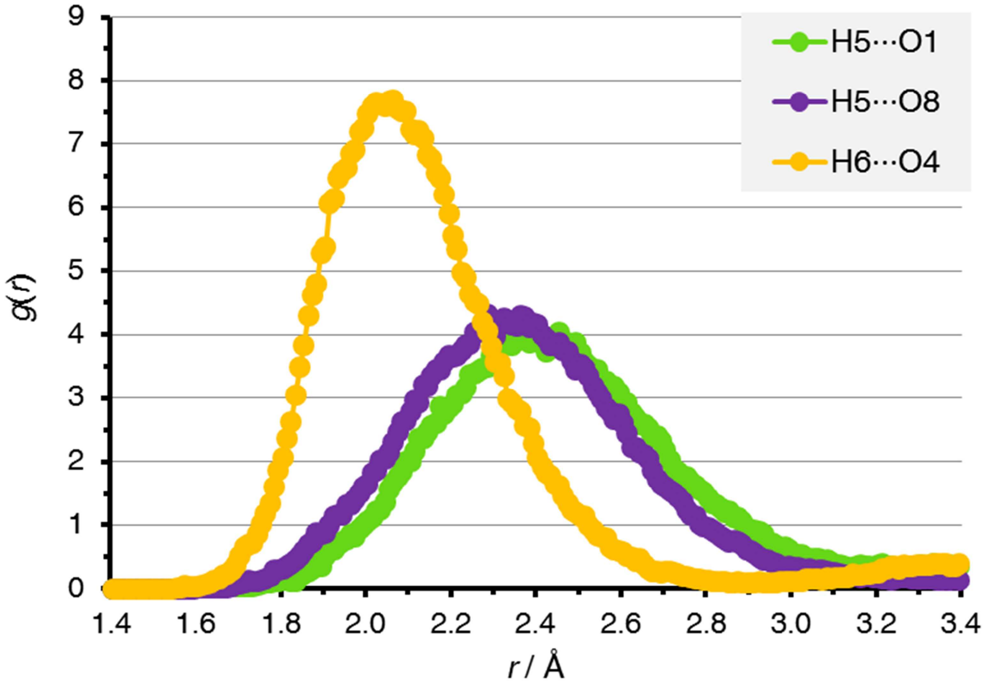

Figure S7: H···O radial distribution functions for the H_2O(pore) molecule.

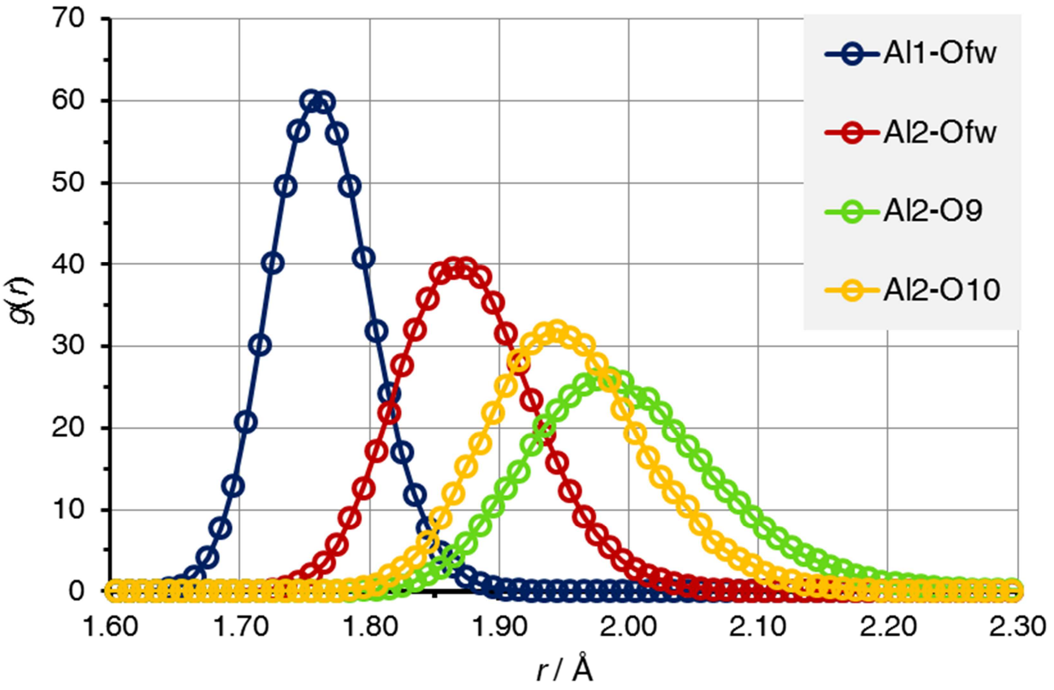

Figure S8: Al-O radial distribution functions. O_{fw} designates framework oxygen atoms.

References

1. Pluth, J.J.; Smith, J. V Hydrated aluminophosphate ($AlPO_4.1·5H_2O$) with PO_4, AlO_4 and $AlO_4(H_2O)_2$ groups and encapsulated water. *Acta Crystallogr. Sect. C Cryst. Struct. Commun.* **1986**, *42*, 1118–1120.

2. Keller, E.; Meier, W.M.; Kirchner, R.M. Synthesis, structures of $AlPO_4$-C and $AlPO_4$-D, and their topotactic transformation. *Solid State Ionics* **1990**, *43*, 93–102.
